# From service provision to function based performance - perspectives on public health systems from the USA and Israel

**DOI:** 10.1186/2045-4015-1-46

**Published:** 2012-11-26

**Authors:** Douglas F Scutchfield, Ehud Miron, Richard C Ingram

**Affiliations:** 1 University of Kentucky College of Public Health, 111 Washington Avenue, Lexington, KY, 40509, U.S.A; 2 Israel Association of Public Health Physicians, Nissenboim 4/23, Haifa, 32807, Israel

## Abstract

If public health agencies are to fulfill their overall mission, they need to have defined measurable targets and should structure services to reach these targets, rather than offer a combination of ill-targeted programs. In order to do this, it is essential that there be a clear definition of what public health should do- a definition that does not ebb and flow based upon the prevailing political winds, but rather is based upon professional standards and measurements.

The establishment of the Essential Public Health Services framework in the U.S.A. was a major move in that direction, and the model, or revisions of the model, have been adopted beyond the borders of the U.S.

This article reviews the U.S. public health system, the needs and processes which brought about the development of the 10 Essential Public Health Services (EPHS), and historical and contemporary applications of the model. It highlights the value of establishing a common delineation of public health activities such as those contained in the EPHS, and explores the validity of using the same process in other countries through a discussion of the development in Israel of a similar model, the 10 Public Health Essential Functions (PHEF), that describes the activities of Israel’s public health system. The use of the same process and framework to develop similar yet distinct frameworks suggests that the process has wide applicability, and may be beneficial to any public health system.

Once a model is developed, it can be used to measure public health performance and improve the quality of services delivered through the development of standards and measures based upon the model, which could, ultimately, improve the health of the communities that depend upon public health agencies to protect their well-being.

## Preface

In order for a public health system to be successful in its efforts to improve the health of communities, the entities making up the system must have a clear direction for their actions. Many programs have specific goals, such as reducing smoking or to improve screening rates screening rates. However, it is difficult for the entities tasked with delivering a multitude of these programs to coordinate their efforts, develop an overall strategy to improve the public’s health, or assess where best to direct their actions, without a set of overarching goals, services or functions with which to assess performance and need. The lack of direction, goals, or mutually agreed upon responsibility often results in inefficiency, misdirected resources and likely ineffectual activities. It may also encourage public health agencies to function in programmatic silos, as opposed to engaging in strategic activities that span programs. For these reasons it is imperative, with the decline in many countries of the resources available to the health sector in general and public health specifically, to have clear aims and well defined functions, as well as measures of success.

While the tools of public health are specific and the methodologies used are mostly well-defined and agreed upon, it is clear that, due to the vast scope of public health, attempts to examine the larger picture of public health and perform comparative analysis of systems often get blurred by poorly defined terms of reference, and are adversely affected by lack of agreement on the specific functions of public health. The variations in public health infrastructure, training, and competencies, combined with different vocabularies and terminologies, make cross-national dialogue between public health professionals more difficult and limit the possibility for exchange of best practices among nations.

It is with those limitations in mind that we examine the potential emergence of a new vocabulary at a global level and its impacts on two different public health systems – Israel and the U.S.A. We believe there is potential benefit for other countries interested in joining efforts to clarify the role, scope, and functions of public health.

## U.S.A.

### Delineating essential public health services in the U.S

Early U.S. efforts to delineate the functions of public health distinctively focused on the role of public health in preserving the economic functions of the new nation. Shipping and commerce were key aspects to assuring the economic wellbeing of the United States, and early attempts to protect the public health were directed at those areas. The Marine Hospital Service, one of the first governmental attempts to protect public health in the United States, was developed in the late 1700s with the express purpose of providing for the healthcare of seamen - a key cog in the shipping and trade activities of the United States. Many of the early local boards of health and health departments were located in shipping areas, and played key roles in administering quarantine of ships in harbors [1].

As public health matured in the United States, a movement began to broaden the role of public health, and adopt a more holistic approach to the prevention of disease. One example of this shift was the work of Lemuel Shattuck, a bookseller in the state of Massachusetts. Shattuck was commissioned to examine the state of public health in Massachusetts, and produced the *Report of the Sanitary Commission of Massachusetts* in 1850 [2]. The report recommended a comprehensive set of public health programs and activities that went well beyond providing healthcare for seamen and quarantine. These included child care, environmental health, health education, community planning and numerous other services that governmental public health organizations should provide. Shattuck’s report was largely ignored at the time, but was subsequently used to structure many public health programs and agencies [3-5].

While Shattuck’s report was one of the first known attempts to delineate the functions of public health in the United States, a major milestone was reached in the twentieth century with the 1945 publication of Haven Emerson’s report for the American Public Health Association on Local Public Health Units [6]. This report called for all Americans to be covered by basic public health services, which were defined by the committee on administrative practice and has become known as the “Basic Six” functions: 1. Vital Statistics; 2. Communicable Disease Control; 3. Laboratory Services; 4. Health Education; 5. Maternal and Child Health; and 6. Environmental Health. Emerson’s “Basic Six” services became, over the next several decades, the industry standard for local public health services and the service standard for health departments [3]. Emerson’s report essentially delineated six specific programmatic areas of public health practice.

### The core functions of public health and 10 essential public health services

Another milestone in the development of public health in the United States, and attempts to define the services, functions, and activities provided by public health agencies, grew out of the landmark Institute of Medicine (IOM) report, entitled *The Future of Public Health*, that was published in1988 [7]. The IOM report provided a clear definition of the mission of public health: “creating conditions in which people can be healthy.” The report also asserted that the responsibilities of governmental public health fell into three broad categories, known as the three core functions of public health:

1 Assessment, reflecting the necessity for every health department to collect, assemble, analyze, and communicate information about the health of their community

2 Policy Development, focusing on the responsibility of the health department to identify health problems based on data and to use evidence to implement policies to solve community health problems

3 Assurance, reflecting the responsibility that public health agencies have to ensure that the population that they serve has the programs and services that they require, either through direct provision of programs and services or assuring that others provide those programs and services.

While the report specifically focused on governmental public health, it also recognized that other organizations and agencies contributed to the achievement of these objectives and the public health mission in communities. The report referred to this broader array of partners as the public health system, which, as a concept, was quite different from the more traditional focus on the strict governmental role in the provision of public health [7].

In the 1990s, in part related to efforts to establish health reform in the U.S., it became apparent that the three functions above were overly vague, and did not convey to the public, medical care sector, or most importantly, policy makers the various specific responsibilities of public health departments. While assessment, policy development, and assurance were acceptable they did not clearly delineate the diverse services provided by public health, and the role public health played in disease prevention. As a result, efforts began to further refine and develop a more specific set of core services and functions of public health. Perhaps the most notable of these were the work of the U.S. Centers for Disease Control and Prevention (CDC) Public Health Program Practice Office and the Office of Health Promotion and Disease Prevention, along with several public health organizations and agencies to establish a set of Ten Essential Public Health Services (10 EPHS) that achieved a broad consensus agreement across the public health community[8]. The 10 EPHS are:

1 Monitor health status to identify and solve community health problems.

2 Diagnose and investigate health problems and health hazards in the community.

3 Inform, educate, and empower people about health issues.

4 Mobilize community partnerships and action to identify and solve health problems.

5 Develop policies and plans that support individual and community health efforts.

6 Enforce laws and regulations that protect health and ensure safety.

7 Link people to needed personal health services and assure the provision of health care when otherwise unavailable.

8 Assure competent public and personal health care workforce.

9 Evaluate effectiveness, accessibility, and quality of personal and population-based health services.

10 Research for new insights and innovative solutions to health problems.

In many ways, the EPHS were a logical next step to the movement toward specifically delineating public health services that was started with the work of Shattuck, and advanced by Emerson and the creation of the core functions. Why they took so long to be developed is not clear; this may be a reflection of a shift toward a greater emphasis on chronic disease (where the metrics of success are harder to measure), or it may simply be due to the extreme heterogeneity of public health systems in the United States [9]. While Shattuck and Emerson focused on specific programmatic activities, the core functions and EPHS mark a paradigm shift toward focusing on macro-level services that are adaptable, and can be applied to most of the programmatic areas of public health. The set of 10 EPHS has proven to be useful in clarifying and enhancing the activities of the U.S. public health system. It has gained wide acceptance and is used by practitioners, academics, policy makers, and others engaged in the public health system and its work. The EPHS has been shown to be widely applicable, and has been used for a variety of purposes, including addressing both specific health threats, such as diabetes and workplace safety [10-12], and helping refine agency operations in times of fiscal crisis [13]. In addition, they have been used to assess the delivery of public health services [14,15], and modified and used to improve the delivery of more general public health services, specifically environmental health services [16-18]. Perhaps the most significant impact of the EPHS has been that they transcend the basic programmatic areas contained in the “Basic 6”, and emphasize that it is necessary to focus on population based activities, as opposed to clinical care services, in the provision of public health services. They also reinforce the importance of gathering and using evidence to make decisions related to public health.

While the core functions and EPHS have been useful, to some degree, as a sort of “best practices” guide for public health agencies, their real impact may be that they have facilitated the development of measures of effectiveness of the public health system tied to those services. Specifically identifying the services made it possible to measure the performance of a health department by examining the extent to which they adequately provide the services described in the core functions or ten EPHS. This, in turn, has facilitated a move toward implementing performance management in many public health agencies in the U.S.

### Measuring public health performance

The first attempt to measure public health performance, based on the three core functions, was a joint effort between B.J. Turnock, at the University of Illinois, Chicago, and C. Arden Miller, at the University of North Carolina. They compiled a series of 20 questions designed to measure local public health agency performance in relation to 20 specific activities linked to one of the three core functions; this provided public health agencies with a tool they could use to systematically assess their performance relative to a standard, identify areas of excellence and areas that needed improvement, and allocate resources accordingly [19,20]. This early method is still used in some quarters to measure local health department performance.

**Figure 1:**
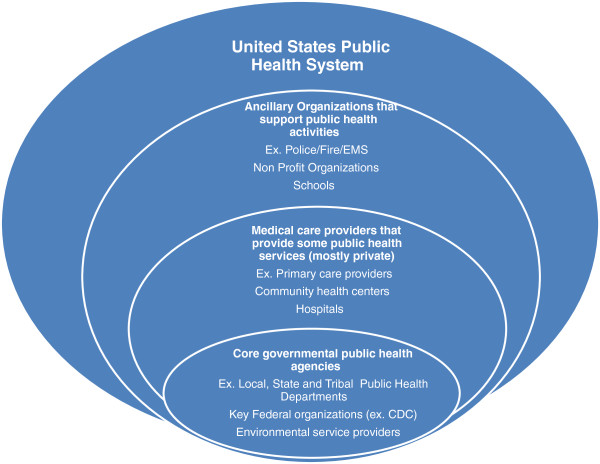
United States Public Health System.

In the late 1990s, there was another, more sophisticated effort to measure public health performance, this time based on the ten EPHS, and focused on the public health system. One key aspect of the 1988 IOM Report was that it addressed both governmental public health agencies and the larger public health system, defined as all the organizations, agencies, and actors who contribute to the mission of public health [7]. This includes schools, media, other governmental agencies, clinical service providers, and non-profit agencies, which are a part of the effort of assuring the population receives the public health services needed. Figure 1 is a graphical representation of the U.S. public health system.

The CDC, working with a number of other agencies, developed a program to measure the performance of the public health system relative to the 10 EPHS- the National Public Health Performance Standards Program [21]. The program was responsible for the development of three new instruments to measure public health performance in local public health systems, state public health systems, and local public health governance organizations. These measures were validated and have been used for a number of purposes: quality improvement, assessment of community health capacity, and research on the public health system [12,14,15,21-24].

### Performance measurement as a basis for accrediting public health agencies

The long movement toward clearly defining and standardizing public health operations, begun by Shattuck in the 1800s, has recently found expression in the creation of a national voluntary accreditation program, based to some degree on the EPHS, of governmental public health agencies in the U.S. The movement toward accreditation of public health agencies was motivated, in part, by a realization that accreditation frameworks were already in place for many similar governmental enterprises, as well as entities in the health care system, and these entities enjoyed increased credibility as a result. Accreditation of public health agencies has the dual purpose of promoting quality improvement activities and assuring accountability to the public for the public monies expended. While the “Basic Six”, the core functions and the 10 EPHS were all attempts to tell public health agencies what should be done, accreditation seeks to ensure that they use best practices to do it. The Public Health Accreditation Board (PHAB), a non-profit entity, has been working over the last several years to establish this voluntary public health accreditation system, which started accepting applications for accreditation in the fall of 2011 [25,26]. One major challenge faced by PHAB was developing standards and measures that were specific enough to be meaningful, while being general enough to encompass the large amount of heterogeneity in public health in the U.S., where each community served by a public health agency is unique, and has differences that must be accounted for in the services and programs of the health department. Fortunately, two frameworks that could be easily adapted to assess agency function already existed- the 10 EPHS, and National Public Health Performance Standards Program [27]. It seems apparent that agreement on a set of standards and functions that all health departments should provide or perform has much utility. As the examples of the 10 EPHS, the NPHPSP and PHAB accreditation suggest, these standards and functions have utility, and can be used to guide agency operations, improve the delivery of specific services, and measure the performance of the entire public health system.

While the set of services established in the U.S. may not be the standard that would serve every country, the mechanism used to establish these services may serve as a model that can be adapted to create a set of services specific to a particular nation. The mechanism used in the U.S. allows for accountability, transparency, mechanisms for quality assurance and improvement, and demonstration of the role and responsibility of this often misunderstood and neglected unit of government.

## Israel

### The development of public health services

Starting the late 19^th^ century, several Jewish voluntary organizations were concerned with providing health services, including public health services (specifically disease prevention and health promotion), to the Jewish community in Palestine. The major providers were the Workers' Sick Fund (a sort of health maintenance organization established by the General Federation of Labor) and the Hadassah Medical Organization (established by a U.S. based Jewish voluntary organization, and focused on providing medical care and education) [28]. The public health services provided by the two organizations included vaccinations, mother and child health clinics, and health promotion. The Hadassah Medical Organization was particularly focused on improving maternal and child health, and its first foray into providing medical care was in 1913 through a program known as “Tipat Halav”, that supplied milk to mothers unable to nurse. This program later grew into a series of clinics, fully funded by the state, that provide maternal and child preventive health services [28,29].

In 1917, after the British army occupied Palestine, the British administration published Public Health Ordinance No. 1, regulating the general health service of the country. The ordinance covered a variety of areas, including the practice of medicine, the registration of infectious diseases, births and deaths, vaccinations, burials, and general sanitation. The ordinance was followed by additional legislative and administrative regulations encompassing quarantine regulations, pharmacy, anti-malarial ordinances, water sanitation, and more [30-32]. The 1940 version of the British administration's Public Health Ordinance was preserved in the Israeli legal system, and the public health services provided by both the British Health Services in Palestine and the local medical providers were integrated in a branch of the Israeli Ministry of Health (MOH) - the Public Health Services (PHS) [28,33].

In 1995, Israel implemented a National Health Insurance Law (NHIL), defining the basket of personal health services that would be provided by the Health Maintenance Organizations (HMOs) and the government to reach Israel’s population. These services include the provision of mother and child health services, and school health services [28,33]. The two latter services were to be provided by the government as stipulated in the third amendment to the law.

The history of public health development in Israel is relevant to the way the different functions contained in the EPHF have been delivered in the Israeli Health System and the nature of their providers.

### Public health services (PHS) within the ministry of health (MOH)

The PHS today is a major division within the MOH, and it is charged with implementing the items specified in the Public Health Ordinance, as well as some personal preventive health services specified in the third amendment to the NHIL [28]. The PHS operates at regional levels through 7 Regional Health Offices (RHO) and, also, at district levels in three regions through 13 District Health Offices (DHO). The RHO and DHO infrastructure is comparable to city or county level agencies in the U.S. that operate as divisions of the state level agencies. The RHOs and DHOs function as MOH branches, with governance authority and statutory powers derived from the Public Health Ordinance and other public health laws, such as the Business Licensing Law, the Environmental Protection Law, and the National Health Insurance Law. Both RHOs and DHOs have statutory powers in implementing quarantines or taking measures to safeguard public health based on the provisions in the Public Health Ordinance and the Business Licensing Law. The major difference between the two levels is in the larger range of functions that the RHOs are required to perform, including supervisory authority over the activities of health providers (HMOs, hospitals, dental services, etc.).

The PHS does much work, particularly as it relates to the provision of personal preventive health services, through the previously mentioned network of approximately 500 Mother and Well Child Clinics created on the basis of the early 20^th^ century original Hadassah sponsored clinics. These Mother and Well Child Clinics provide ante-natal care and follow-up, family planning activities, and post-natal care to the infants and their mothers, including health promotion, childhood vaccinations, and screening for developmental disorders.

### The public health system

The PHS is not the only member of the public health system in Israel. The four HMOs operating in Israel provide personal preventive health services and, to some extent, community health promotion services. The HMOs provide primary health care on the basis of the 1995 NHIL, which specifies a basket of services that are provided to the general population and funded by a health tax imposed on all citizens, according to income, and collected by the National Insurance Institute. The money is passed on to each HMO using a capitation formula based on membership, and the age distribution of membership in the HMO. Deficits in HMO funding are covered with direct funding from the Ministry of Finance [28].

**Figure 2:**
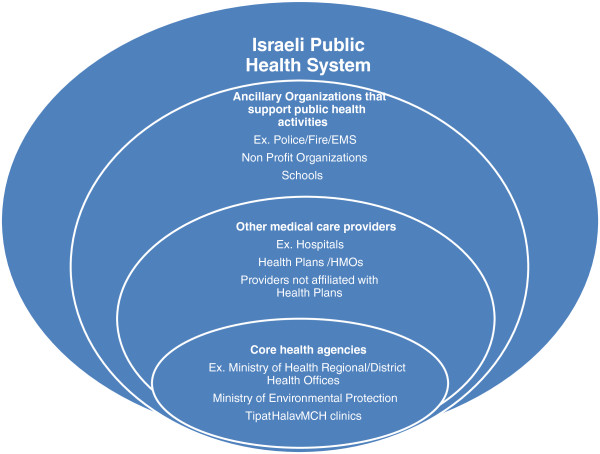
Israeli Public Health System.

Figure 2 is a graphical representation of the Israeli public health system. The figure, much like Figure 1, suggests the complexity associated with a public health system that extends beyond the governmental elements involved in public health, and beyond the medical elements classically associated with health. It is quickly apparent that the major components of a public health system are similar between countries, with minor alterations reflecting local variation. In this case, there is clearly greater integration between medical care and public health, and a less defined distinction between private medical care agencies and governmental public health agencies in Israel.

Much of the ongoing debate about public health in Israel has concentrated on what services the state should provide to the general population. [28] Unfortunately, this debate has not focused on the public health system as a system in which the stakeholders can and should cooperate to deliver a specified and definable set of services, or fulfill a defined set of public health population functions. Decisions about changes in services and activities of the public health department (or the PHS in Israel) have often occurred without an over-arching set of strategic plans, goals, or mission, but on an ad hoc basis, driven by funding, rather than a rational consideration of the role and responsibility of governmental public health or, for that matter, the general public health system [34]. A shift of focus from debating the provision of specific services, and the ideologically biased outlooks on financing public health, to an outlook on the public health system as a function oriented system would allow a much needed review of the functions as currently performed and compared with optimal function and their level of performance.

This need for a systems approach and an evidence-based review of performance was recognized by the Israel Association of Public Health Physicians (IAPHP) and also by several senior Israeli health services researchers. The IAPHP is part of the Israeli Medical Association (IMA) and is the leading authority on public health issues in the IMA. Due to the powerful impact of the IMA on the Israeli health system the importance of IAPHP recommendations upon general IMA policy is significant. Furthermore, many of the IAPHP members hold positions of influence in the health system and their combined endorsement may affect the adoption of new guidelines and policies by the Ministry of Health.

The IAPHP took the lead in attempting to develop a series of essential public health services/functions, known as the Public Health Essential Functions, which could achieve consensus as a set of responsibilities for the Israeli public health system at national, regional and district levels. Much like the EPHS in the U.S., the framework of the Public Health Essential Functions can serve as a basis for public health performance measurement. It was developed and adopted by the IAPHP, as described below.

The IAPHP began with a review of The Pan American Health Organization (PAHO) model of essential public health functions. The PAHO model, in turn, had benefitted from the previously discussed U.S. list of EPHS. The IAPHP felt that the PAHO modifications were a better starting point than the EPHS, since the PAHO model is tailored for public health systems which operate in a non-federal environment, and where there is a central authority responsible for public health with authority decentralized to varying degrees according to specific country needs. The PAHO model, originally derived from the State-Level EPHS model created by the CDC, was used as the source for a set of 11 functions (later narrowed to 10) that describe the perceived functions of the public health system in Israel. The set of functions was defined by several workgroups of public health professionals, and further validated with the help of senior officials in the Israeli health system.

It should be noted that the shift in terminology from "Services" to "Functions" was deemed necessary to better differentiate the model from the PHS unit operating in the MOH, and avoid any confusion between the functional unit and the tasks which it may perform. A second reason for the modification is the need to shift the debate to targets and standards of performance rather than who provides a specific service within the public health system.

The IAPHP adopted the set of essential functions derived from the process described above, and deemed that this set of essential functions should provide the underpinning for revising the responsibilities and expectations of the Israel PHS. The IAPHP also felt this proposal allows for alignment of public health infrastructure, and better definition of activities assumed by both the governmental PHS and the broader public health system. The 10 Essential Functions of the Israel public health system adopted by the IAPHP are:

1. Leading, planning and developing public health policy and management of the health system according to policy.

2. Monitoring and evaluation of population health status with the aim of identifying situations which require intervention: health related needs, health related risks and inequalities in health

3. Evaluation of efficiency, effectiveness and quality of individual and community health services

4. Identification, prediction, prevention and control of environmental related health risks (habitat, workplace, air, food and water)

5. Initiation, promotion and carrying out of health related research

6. Health promotion and disease prevention.

7. Preparedness for and mitigation of unusual events which have an impact on public health: disease outbreaks, abnormal morbidity and natural or men-made disasters

8. Development and training of public health workforce

9. Creating partnerships to promote knowledge, coordination and optimal use of resources

11. Promoting legislation, control and enforcement or laws and regulations in public health

The list of Israeli functions has been endorsed by the IMA, and efforts are currently underway to secure the endorsement of the Israeli Ministry of Health as well as other governmental and non-governmental organizations using focus groups and open-table discussions.

Much as delineating a list of essential public health services facilitated the development of performance measurement tools for the U.S. public health system, developing a set of essential functions has facilitated the development of a pilot performance measurement tool for the Israeli public health system. The National Public Health Performance Standards Program’s local public health system instrument developed to measure the U.S. system has been adapted to create a new instrument, designed to measure the performance of the essential functions in Israel. This instrument has been used in a pilot test to determine its ability to measure performance in the Northern Regional Office of the PHS. This joint project between The University of Kentucky and the Northern Israel Regional PHS Office will be reported on in a separate paper, but it does show the utility of going through the process of establishing a set of agreed upon public health functions specific to a particular country, that can be later used to both measure and improve performance, and improve community health.

The effect on the infrastructure of the public health system of adopting an EPHF-like system is difficult to estimate; it may well result in a major structural reform or focus on milder incremental changes. It is part of a long-term process in which the EPHF system is just a first step leading to additional steps in standardization, training and accreditation. In Israel the suggestions regarding structural change range from no change necessary to the establishment of a Public Health Institute dedicated to investigating topics including optimal system structure and methods of service delivery, a ministerial committee on public health and even the establishment of a public health ministry. At this stage, the required resource allocation cannot be estimated nor the eventual changes.

## Conclusions

This paper describes several key steps in the attempts to delineate essential public health services or functions in the U.S. and Israel. It illustrates that, regardless of history, eventually public health systems may become so complex that it is useful to develop and describe a set of functions that can and should be expected of a public health department or system. While some national initiatives, such as the various “Healthy People” initiatives in the U.S. or Healthy Israel 2020 can be used by local health entities to set goals and targets for performance, they are still largely programmatic in nature, and reflect national goals. Thus, they may not be a reflection of the needs of a specific locality, or encompass the broad scope of public health activities. As a result they may not be useful for directing day-to-day operations in public health entities. These lists of functions may also be used to promote activities intended to improve the quality of public health service delivery in different types of public health systems, through initiatives such as the development of tools to ascertain the performance of the public health department and system.

This effort illustrates the value of international cooperation in attempting to define, describe, and examine public health systems regardless of how they are established and structured. We believe that this work provides an illustration of how other countries might go about the work of duplicating (with appropriate adaptation to local circumstances) our own efforts in the U.S. and Israel. We believe that the opportunity for international comparisons and activities in public health has value and would benefit those who wish to pursue this idea. The experiences in the U.S. and Israel suggest that technology and programs can be adopted by different countries, and show the methods that can be used to adapt them to different public health environments.

We believe that the World Health Organization would benefit by taking on the responsibility for attempting to further emulate our experience in bi-national collaboration to develop and use well-defined functions for health departments. Public health is becoming an increasingly global endeavor, and, given revolutionary changes in transportation and information sharing, public health concerns are beginning to span national boundaries and assume a more international scope. In addition, the challenges faced by public health agencies are becoming more complex with the shift in many regions from focusing solely on infectious disease to also addressing chronic disease. As a result, the solutions developed by public health agencies are becoming more complex as well. Having clearly defined functions of the public health system can help guide the coordination and delivery of the multitude of programmatic services that may be necessary to address a single problem. The dynamic nature of this new public health suggests a need for guidelines specific enough to guide practice, but general enough in scope to facilitate the delivery of a multitude of services. Given this new reality in public health, using uniform, but flexible methods, to develop well defined functions for public health departments may be a key aspect of attempts to develop guidelines that assure that the health of all the nations is protected.

## Competing interests

The authors declare that they have no competing interests.

## Authors’ contributions

EM and FDS collaborated on developing the conceptual framework for this manuscript, and have played key roles in the development of the EPHS in the US, and the EPHF in Israel, and applied that experience to writing this manuscript. RI contributed to the development and refinement of the ideas in the manuscript. All authors contributed to the writing of this manuscript, read the final draft, and approved the final draft.

## Authors’ information

F. Douglas Scutchfield is the Peter P Bosomworth Professor of Health Services Research and Policy at the University of Kentucky Colleges of Public Health and Medicine. He holds an M.D. degree from the University Of Kentucky College Of Medicine, is the Director of the U.S. National Coordinating Center for Public Health Services and Systems Research, and is the principal investigator on a number of research projects examining the U.S. public health system. Ehud Miron is a public health physician, director of the Israeli Ministry of Health’s Nazareth District and chairman of the Israel Association of Public Health Physicians and of the World Federation of Public Health Associations’ Workgroup on Public Health Education and Training. Richard Ingram is a research assistant professor at the University of Kentucky College of Public Health. He holds a Dr.P.H. degree from the University of Kentucky College of Public Health. His research interests include public health delivery system structures and the relationship between variations in system structure and health outcomes.
